# Efficacy and Safety of Response-Adapted Intravitreal Methotrexate Therapy for Vitreoretinal Lymphoma: A Two-Center Retrospective Cohort Study

**DOI:** 10.3390/jcm15187323

**Published:** 2026-09-21

**Authors:** Mihai-Luca Cioboată, Ioana Tofolean, Suher Abduraman, Radu Burcea, Maria-Alexandra Chiotan-Călin, Radu-Alexandru Chiotan, Dana-Margareta-Cornelia Dăscălescu, Miruna Cioboată, Ali Rıza Cenk Çelebi, Florian Baltă

**Affiliations:** 1 Department of Ophthalmology, Carol Davila University of Medicine & Pharmacy, 020021 Bucharest, Romania; mihai-luca.cioboata@drd.umfcd.ro (M.-L.C.); ioana.tofolean@umfcd.ro (I.T.); radustefan505@yahoo.com (R.B.); maria-alexandra.chiotan@drd.umfcd.ro (M.-A.C.-C.); radu.chiotan@umfcd.ro (R.-A.C.); dana.dascalescu@umfcd.ro (D.-M.-C.D.); florian.balta@umfcd.ro (F.B.); 2 Department of Ophthalmology, Clinical Institute of Ophthalmological Emergencies “Prof. Dr. Mircea Olteanu”, 010464 Bucharest, Romania; abduramansuher@yahoo.com; 3 Retina Clinic, 014142 Bucharest, Romania; 4 National Institute of Pneumophthisiology “Marius Nasta”, 050159 Bucharest, Romania; 5 Department of Ophthalmology, Istanbul Medipol University, 34196 Istanbul, Türkiye

**Keywords:** vitreoretinal lymphoma, intravitreal methotrexate, visual acuity, optical coherence tomography, treatment outcome, ocular relapse, intraocular lymphoma

## Abstract

**Objectives:** Longitudinal visual and imaging outcomes after intravitreal methotrexate (MTX) for vitreoretinal lymphoma (VRL), and baseline factors associated with remission, remain poorly defined. We evaluated the 12-month efficacy and safety of a reduced-intensity induction regimen followed by response-guided continuation therapy. **Methods:** Seventeen consecutive patients with VRL confirmed by vitreous cytology and flow cytometry received intravitreal MTX (400 μg/0.1 mL) at two centers in Bucharest, Romania, between 2018 and 2023. Visual acuity (VA), vitritis and optical coherence tomography (OCT) findings were assessed at baseline and at 1, 3, 6 and 12 months (Friedman and Cochran’s Q tests). **Results:** Mean VA improved from 1.22 ± 0.70 to 0.74 ± 0.63 logMAR (χ^2^ = 30.82, *p* < 0.001; Kendall’s W = 0.45), with statistically significant improvement from month 6. At the scheduled 12-month assessment, vitritis and retinal infiltrates had resolved (both unadjusted *p* < 0.001). Sub-RPE lesions and outer retinal hyperreflectivity decreased (both unadjusted *p* = 0.031), but these comparisons did not meet the Bonferroni-adjusted significance threshold. Outer retinal disorganization and foveal involvement did not change significantly. Fourteen patients (82.4%) achieved complete ocular remission (median, 6 months); three (17.6%) relapsed and regained disease control after retreatment. Keratopathy was the most frequent adverse event (41.2%); no severe complications occurred. **Conclusions:** This regimen was associated with a high ocular remission rate, progressive visual improvement and a manageable safety profile. Because all 10 patients with central nervous system lymphoma also received systemic therapy and/or radiotherapy, ocular outcomes cannot be attributed solely to intravitreal MTX.

## 1. Introduction

Intravitreal methotrexate (MTX) is one of the most widely used local therapies for vitreoretinal lymphoma (VRL). This route of administration enables therapeutic intraocular drug concentrations to be achieved while minimizing systemic exposure. However, repeated injections may result in cumulative ocular surface toxicity [1,2,3].

Despite its widespread use, several clinically relevant aspects of intravitreal MTX therapy remain insufficiently understood. In particular, the temporal relationship between visual recovery and clinical or imaging response has not been fully characterized. Moreover, limited evidence is available regarding the regression or persistence of optical coherence tomography (OCT) abnormalities following treatment. Baseline factors associated with complete ocular remission also remain poorly defined [4,5]. Most published studies have focused primarily on overall remission and relapse rates, with relatively few evaluating functional and structural outcomes concurrently at predefined follow-up intervals.

The optimal intensity and duration of intravitreal MTX therapy also remain uncertain. Conventional treatment regimens may involve up to 25 injections administered over approximately one year, resulting in a considerable treatment burden and an increased risk of cumulative ocular surface toxicity. Although reduced-intensity regimens have been proposed, the evidence supporting their efficacy and safety remains limited [6,7]. Therefore, we conducted a retrospective two-center cohort study to characterize changes in visual acuity (VA) and OCT findings over 12 months, determine ocular remission and relapse rates, explore baseline factors associated with complete remission, and assess the ocular safety profile of a reduced-intensity induction regimen followed by response-guided continuation therapy.

## 2. Materials and Methods

### 2.1. Study Design

This retrospective observational study included patients treated between January 2018 and December 2023 at the Clinical Institute of Ophthalmological Emergencies “Prof. Dr. Mircea Olteanu” and the Retina Clinic in Bucharest, Romania.

### 2.2. Patients

Adult patients with cytologically confirmed VRL were eligible for inclusion if they had received at least one intravitreal injection of MTX, irrespective of concomitant treatment, and had completed at least 12 months of clinical and imaging follow-up. Patients were excluded if they had less than 12 months of follow-up, incomplete clinical documentation, or coexisting ocular conditions that could substantially confound the interpretation of VA or OCT findings. Seventeen patients met the eligibility criteria.

The diagnosis was established by cytological examination of vitreous biopsy specimens, supported by flow cytometric immunophenotyping and interpreted in conjunction with the clinical and imaging findings. Other ancillary diagnostic techniques, including immunocytochemistry, intraocular cytokine analysis (IL-10/IL-6 ratio), immunoglobulin heavy-chain gene rearrangement analysis, and MYD88 L265P mutation testing, were unavailable at the participating centers. The implications of restricting inclusion to cytologically confirmed cases are addressed in the Discussion section.

At diagnosis, 13 patients (76.5%) had bilateral ocular involvement, whereas 4 (23.5%) had unilateral disease. During follow-up, contralateral ocular involvement developed in two of the four patients who initially presented with unilateral disease. Consequently, 15 patients (88.2%) had bilateral involvement by the end of the follow-up period. Outcomes were not compared between unilateral and bilateral cases because only four patients initially had unilateral disease. In patients with bilateral involvement, the study eye was defined as the eye with worse VA at treatment initiation.

### 2.3. Treatment Protocol

All patients received intravitreal MTX at a dose of 400 μg/0.1 mL according to a standardized institutional protocol comprising a fixed initial phase of seven injections. This phase consisted of four weekly injections during induction, followed by two biweekly injections during consolidation and one monthly injection during maintenance.

Following the fixed initial phase, subsequent treatment was guided by clinical and imaging responses rather than by a predetermined total number of injections. At each scheduled assessment, the multidisciplinary team determined whether treatment should be continued, temporarily withheld, or resumed based on the persistence of vitritis, active retinal or sub-RPE lesions on OCT (Topcon Corporation, Tokyo, Japan), ocular relapse, and treatment tolerability. Accordingly, the total number of injections reflected the individual disease course and did not represent a uniformly abbreviated regimen.

This approach differs from the conventional regimen of approximately 25 injections administered over one year according to a predefined schedule: twice weekly for 4 weeks, weekly for 8 weeks, and monthly for 9 months, irrespective of the timing of clinical response [6]. In the present protocol, the induction phase was less intensive, with weekly rather than twice-weekly injections, and the duration of subsequent treatment was determined by the individual response. We therefore describe this approach as a reduced-intensity induction regimen followed by response-guided continuation therapy rather than as a uniformly shortened treatment course.

### 2.4. Assessments

Patients underwent ophthalmological evaluation before treatment initiation and at approximately 1, 3, 6, and 12 months. At each assessment, best-corrected VA, vitritis grade, the presence of retinal infiltrates, and OCT features—including sub-RPE lesions, outer retinal hyperreflectivity, outer retinal disorganization, foveal involvement, and subretinal fluid—were recorded.

For the exploratory remission analysis, a baseline composite OCT score was calculated by assigning one point for each of six findings: retinal infiltrates, sub-RPE lesions, outer retinal hyperreflectivity, outer retinal disorganization, foveal involvement, and subretinal fluid. The score ranged from 0 to 6, with higher values indicating a greater baseline imaging burden.

Visual acuity measurements were converted to logarithm of the minimum angle of resolution (logMAR) units. Counting fingers and hand-motion visual acuities were assigned values of 1.9 and 2.3 logMAR, respectively. In patients with bilateral involvement, one study eye—the eye with worse VA at treatment initiation—was selected and followed longitudinally throughout all analyses.

Complete ocular remission was defined as complete resolution of vitritis on clinical examination together with the absence of active retinal or sub-RPE lymphoma lesions on fundus examination and serial OCT. Active lesions were adjudicated by persistent, newly appearing, or enlarging infiltrative hyperreflective material in the retinal or sub-RPE compartments, particularly when accompanied by vitreous inflammation or corresponding funduscopic infiltrates. Stable outer retinal disorganization, ellipsoid-zone loss, or RPE irregularity without progressive infiltrative material or vitritis was classified as an inactive structural sequela and did not, in isolation, preclude remission. Remission was adjudicated from the combined clinical examination, fundus findings, serial OCT evolution, and response to treatment rather than from the presence or absence of any single OCT feature.

As neuroimaging and cerebrospinal fluid analyses were not performed at the participating ophthalmology centers, information regarding CNS involvement was obtained retrospectively from the oncology service responsible for systemic patient management. Central nervous system status was available for all 17 patients and was recorded as a binary variable indicating the presence or absence of documented CNS lymphoma. However, the timing of CNS diagnosis in relation to the initial ocular presentation could not be reliably established. Therefore, the temporal sequence of ocular and CNS involvement could not be determined, and the occurrence or progression of CNS disease during follow-up was not included among the study outcomes.

### 2.5. Statistical Analysis

Statistical analyses were performed using IBM SPSS Statistics, version 26.0 (IBM Corp., Armonk, NY, USA). Continuous variables were reported as the mean ± standard deviation (SD) or median and interquartile range (IQR), as appropriate. Categorical variables were presented as frequencies and percentages. Changes in continuous variables over time were analyzed using the Friedman test; Kendall’s W was reported as the corresponding effect size. Post hoc comparisons were performed using Wilcoxon signed-rank tests with Bonferroni correction. Changes in categorical variables over time were evaluated using Cochran’s Q test, followed by McNemar tests. The seven baseline-to-12-month McNemar comparisons were considered exploratory and are reported as unadjusted *p*-values; for interpretation after Bonferroni correction, the significance threshold was 0.05/7 = 0.0071. Associations between baseline characteristics and complete ocular remission were assessed using the Mann–Whitney U test for continuous variables and Fisher’s exact test for categorical variables. All tests were two-sided, and *p* < 0.05 was considered statistically significant except where a multiplicity-adjusted threshold is specified.

### 2.6. Ethics

The study was conducted in accordance with the Declaration of Helsinki and applicable national data protection regulations. The study protocol was approved by the Ethics Committee of the Clinical Institute of Ophthalmological Emergencies “Prof. Dr. Mircea Olteanu”, Bucharest, Romania (approval no. 1289/dated 15 February 2026). All patient data were anonymized before analysis.

### 2.7. Use of Generative AI and AI-Assisted Technologies

During the preparation of this manuscript, the authors used ChatGPT (GPT-5; OpenAI, San Francisco, CA, USA) and Claude (Fable 5; Anthropic, San Francisco, CA, USA) for language editing, improving clarity and organization, and assisting with the preparation and formatting of figures and schematic diagrams. These tools were not used to generate or analyze patient data, perform statistical analyses, identify references, or formulate scientific conclusions. No identifiable patient information was entered into either platform. The authors reviewed and verified all AI-assisted content and take full responsibility for the final manuscript.

## 3. Results

### 3.1. Clinical and Treatment Characteristics of the Study Cohort

Seventeen patients treated with intravitreal MTX who completed 12 months of follow-up were included in the analysis. The median number of intravitreal MTX injections was 16 (range, 12–25). Ten patients (58.8%) received additional treatment: seven received high-dose systemic MTX, one received rituximab–methotrexate–procarbazine–vincristine (R-MPV), one received the MATRix regimen, and one underwent radiotherapy. Overall, nine patients (52.9%) received systemic therapy, one (5.9%) received radiotherapy, and the remaining seven (41.2%) received intravitreal MTX alone (Table 1).

**Figure 1:**
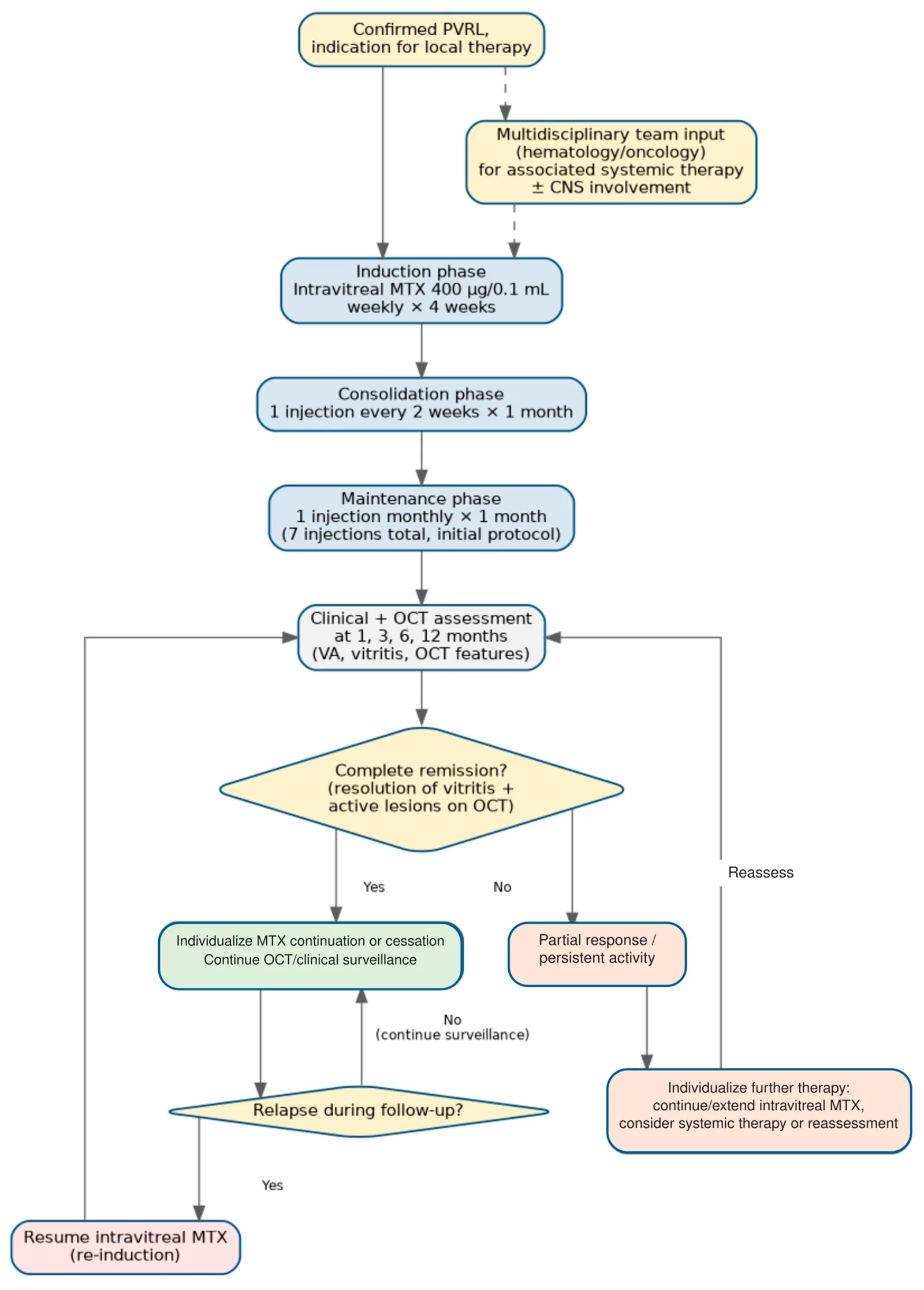
Intravitreal methotrexate treatment and follow-up protocol used in the study cohort. After the initial seven-injection phase, decisions to continue, temporarily withhold, or resume treatment were individualized according to clinical and imaging findings. PVRL, vitreoretinal lymphoma; MTX, methotrexate; OCT, optical coherence tomography; VA, visual acuity.

The intravitreal methotrexate treatment and monitoring protocol is summarized in Figure 1.

Central nervous system lymphoma was documented in 10 patients (58.8%), whereas the remaining seven (41.2%) had no documented CNS involvement. All 10 patients with CNS lymphoma received systemic therapy or radiotherapy, while all seven patients without CNS involvement received intravitreal MTX alone. Consequently, CNS status and the administration of additional treatment overlapped completely and could not be evaluated as independent variables.

No statistically significant differences in ocular outcomes were observed between the two groups. Complete ocular remission was achieved by 7 of 10 patients with CNS lymphoma and by all 7 patients without documented CNS involvement (Fisher’s exact test, *p* = 0.228). Ocular relapse occurred in 2 of 10 and 1 of 7 patients, respectively (*p* = 1.000). Median baseline VA was 1.15 logMAR (IQR, 0.79–2.30) in patients with CNS involvement and 1.00 logMAR (IQR, 0.58–1.00) in those without CNS involvement (*p* = 0.286). At 12 months, the corresponding values were 0.70 logMAR (IQR, 0.30–1.30) and 0.52 logMAR (IQR, 0.16–0.70), respectively (*p* = 0.195). Both groups received a median of 16 injections and had a median time to remission of 6 months (*p* = 0.525). Given the small group sizes and limited statistical power, these findings are reported for descriptive purposes and should not be interpreted as evidence of equivalence between the groups. Similarly, the analyses presented in Section 3.5 should be regarded as exploratory.

### 3.2. Visual Acuity Outcomes During Follow-Up

**Figure 2:**
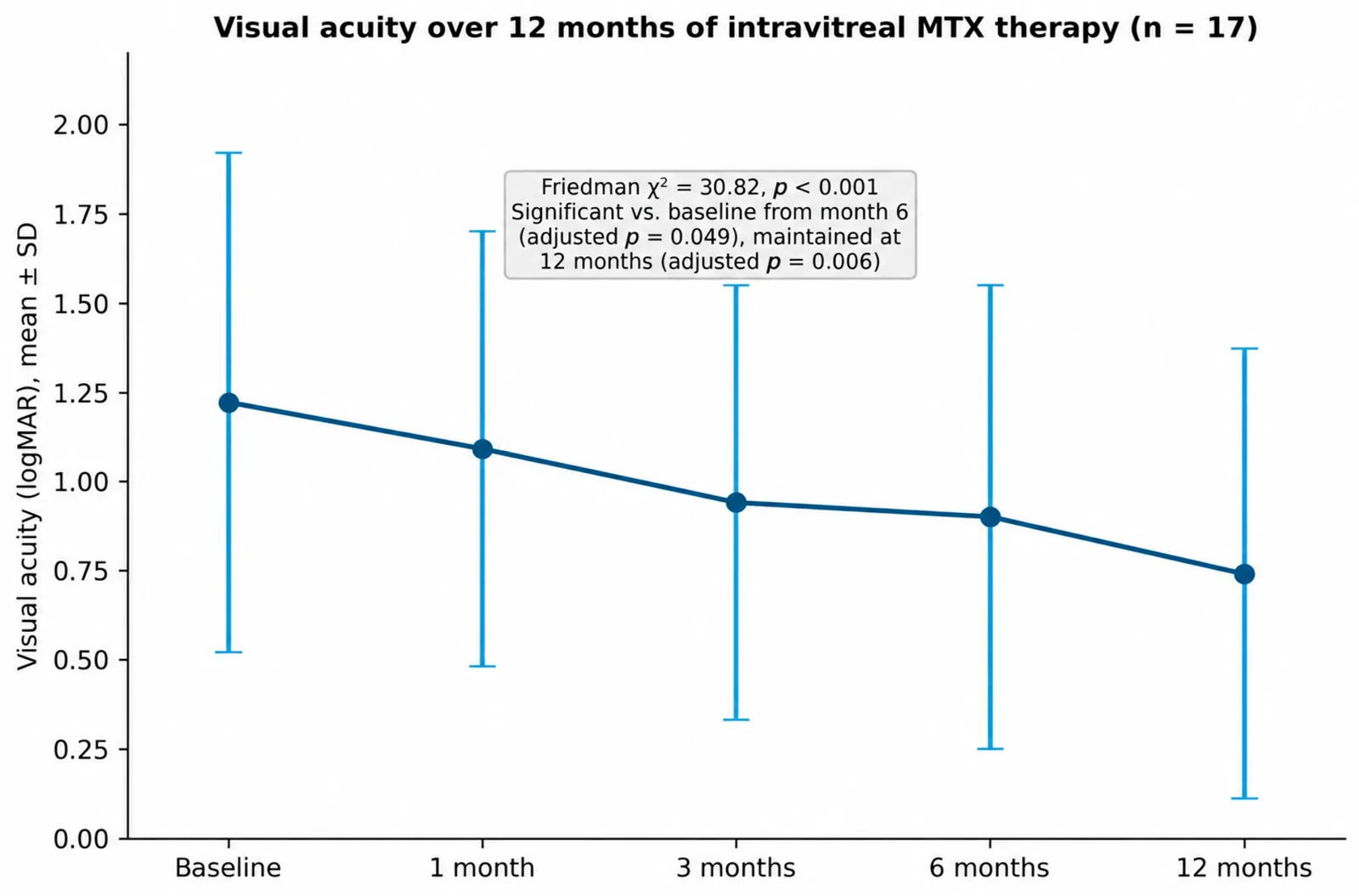
Longitudinal changes in visual acuity during the 12-month follow-up period (*n* = 17). Data points represent mean logMAR VA, and error bars indicate ±1 SD. Lower logMAR values correspond to better VA.

Mean VA improved progressively from 1.22 ± 0.70 logMAR at baseline to 1.09 ± 0.61 at 1 month, 0.94 ± 0.61 at 3 months, 0.90 ± 0.65 at 6 months, and 0.74 ± 0.63 at 12 months (Figure 2). The Friedman test demonstrated a statistically significant change in VA over time (χ^2^ = 30.82, *p* < 0.001), with a moderate-to-large effect size (Kendall’s W = 0.45). Post hoc comparisons using the Wilcoxon signed-rank test with Bonferroni correction showed that the improvement from baseline became statistically significant at 6 months (adjusted *p* = 0.049) and remained significant at 12 months (adjusted *p* = 0.006). Improvements observed at 1 and 3 months did not reach statistical significance after adjustment for multiple comparisons.

At the 12-month assessment, 12 patients (70.6%) demonstrated an improvement of at least 0.2 logMAR, whereas 5 patients (29.4%) remained functionally stable. No patient experienced a deterioration exceeding 0.2 logMAR.

### 3.3. Changes in Vitritis and OCT Features

Vitritis was present in 16 of the 17 patients (94.1%) at baseline. Its prevalence progressively decreased to 7 patients (41.2%) at 1 month, 3 (17.6%) at 3 months, 2 (11.8%) at 6 months, and none at the scheduled 12-month assessment. This reduction was statistically significant (Cochran’s Q = 40.30, *p* < 0.001). Post hoc pairwise comparisons using McNemar’s test demonstrated reductions from baseline at all subsequent scheduled assessments (all unadjusted *p* ≤ 0.004).

Retinal infiltrates were identified in 12 patients (70.6%) at baseline and were absent at the scheduled 12-month assessment (unadjusted *p* < 0.001). Over the same period, the prevalence of sub-RPE lesions decreased from 76.5% (13 patients) to 41.2% (7 patients; unadjusted *p* = 0.031), while outer retinal hyperreflectivity decreased from 70.6% (12 patients) to 35.3% (6 patients; unadjusted *p* = 0.031). The latter two comparisons did not meet the Bonferroni-adjusted significance threshold of *p* < 0.0071 and should be interpreted as nominal, exploratory reductions.

In contrast, no statistically significant changes were observed in outer retinal disorganization (58.8% at baseline versus 41.2% at 12 months; *p* = 0.250) or foveal involvement (47.1% versus 41.2%; *p* = 1.000). Subretinal fluid was present in one patient (5.9%) at both baseline and 12 months, with no change over time (*p* = 1.000) (Table 2).

### 3.4. Ocular Remission and Relapse Outcomes

Complete ocular remission was achieved in 14 of the 17 patients (82.4%) within the 12-month follow-up period, while the remaining three patients (17.6%) did not meet the predefined criteria for complete remission. Among patients who achieved remission, the median time to complete remission was 6 months; timing data were available for 13 of the 14 responders. Most complete remissions occurred within the first 6 months of treatment.

Ocular relapse occurred in three patients (17.6%) at approximately 8, 10, and 12 months after treatment initiation, respectively. In all cases, relapse was characterized by recurrent vitritis and active retinal infiltrates identified through clinical examination and OCT imaging. These event-based relapses were detected at interim visits between the predefined assessments; the last occurred close to, but before, the scheduled 12-month assessment. Resumption of intravitreal MTX resulted in renewed ocular disease control before the scheduled 12-month assessment in all three patients. Thus, the absence of vitritis and retinal infiltrates in Table 2 reflects cross-sectional status at the scheduled 12-month visit and does not exclude relapse earlier during follow-up.

### 3.5. Exploratory Factors Associated with Complete Ocular Remission

Patients who achieved complete ocular remission had significantly better VA at baseline than those who did not (Mann–Whitney U test, *p* = 0.047). A lower baseline OCT score also tended to be associated with complete remission, although this association did not reach statistical significance (*p* = 0.067). Neither the total number of intravitreal MTX injections (*p* = 0.945) nor the administration of additional systemic therapy or radiotherapy was significantly associated with complete ocular remission (Fisher’s exact test, *p* = 0.228) (Table 3).

### 3.6. Ocular Safety

Twelve of the 17 patients (70.6%) experienced at least one ocular adverse event during treatment. Keratopathy was the most frequently reported event, occurring in seven patients (41.2%), followed by transient elevation of intraocular pressure in three patients (17.6%) and conjunctival hyperemia in two patients (11.8%). No serious ocular complications, such as endophthalmitis, retinal detachment, or intraocular hemorrhage, were observed. No patient permanently discontinued treatment because of an adverse event. Most events were transient and were successfully managed with conservative measures or by extending the interval between injections (Table 4).

## 4. Discussion

In this two-center retrospective cohort, a reduced-intensity intravitreal MTX induction phase followed by response-guided continuation was associated with progressive visual improvement, a high rate of complete ocular remission, and acceptable ocular tolerability. Mean VA improved from 1.22 logMAR at baseline to 0.74 logMAR at 12 months, while vitritis and retinal infiltrates resolved completely. In contrast, several structural OCT abnormalities persisted despite clinical remission. Complete ocular remission was achieved in 82.4% of patients, ocular relapse occurred in 17.6%, and all relapsing patients regained disease control after intravitreal MTX was resumed.

The progressive improvement in VA became statistically significant at 6 months and was maintained at 12 months. Furthermore, 70.6% of patients gained at least 0.2 logMAR, whereas none experienced clinically meaningful visual deterioration. These findings suggest that functional recovery may occur gradually and may lag behind the initial resolution of vitreous inflammation and retinal infiltration. Previous studies have reported variable visual outcomes following intravitreal MTX. Zhou et al. [8] observed an improvement in mean VA from 0.65 to 0.30 logMAR, whereas Anthony et al. [9] reported stable VA during the first 12 months despite achieving a favorable local response in most patients. In a larger longitudinal cohort, Kim et al. [10] found a significant improvement in mean VA and identified baseline VA and retinal or subretinal infiltration as independent predictors of long-term visual outcomes. The comparatively poor baseline VA in our cohort may partly explain the delayed functional improvement. Visual recovery in VRL is influenced not only by the resolution of active lymphoma but also by the extent of pre-existing photoreceptor, outer retinal, and RPE damage. Consequently, anatomical disease control may not result in immediate or complete restoration of visual function.

Vitritis decreased markedly within the first month and was absent at the scheduled 12-month assessment. Retinal infiltrates showed a similarly favorable response. These two reductions remained statistically significant after Bonferroni adjustment and support the effectiveness of intravitreal MTX in controlling active inflammatory and infiltrative manifestations of VRL. Optical coherence tomography abnormalities, however, demonstrated different response patterns. Sub-RPE lesions and outer retinal hyperreflectivity showed nominal reductions but did not remain statistically significant after adjustment for the seven exploratory comparisons. Outer retinal disorganization and foveal involvement did not change significantly. Persistent findings in clinically inactive eyes may reflect irreversible structural damage rather than residual lymphoma activity.

Jiang et al., in a study of 111 eyes with cytologically confirmed VRL, similarly found that active infiltrative abnormalities regressed after intravitreal MTX, whereas ellipsoid-zone disruption and RPE abnormalities could persist after treatment [11]. These observations emphasize the importance of distinguishing active infiltrative lesions from post-treatment structural sequelae. Persistent outer retinal disorganization in an eye without vitritis or progressive infiltrative lesions should therefore be interpreted cautiously and should not, in isolation, be considered evidence of active disease [11]. Serial OCT remains valuable for monitoring treatment response, particularly when findings are interpreted in conjunction with VA, vitreous inflammation, fundus appearance, and the evolution of lesions over time [12]. Quantitative OCT parameters and standardized imaging criteria may improve the distinction between persistent structural damage and recurrent lymphoma in future studies [13].

Complete ocular remission was achieved in 14 patients (82.4%), with a median time to remission of 6 months. This rate is consistent with previous reports demonstrating complete response rates of approximately 80% or higher following intravitreal MTX. Anthony et al. [9] reported complete response in 80% of patients at the final assessment, while the large series of Habot-Wilner et al. [14] confirmed effective local disease control in most treated eyes. The seven-injection initial phase used in our cohort was comparable to the modified regimen proposed by Zhou et al. [7], consisting of four weekly injections, two biweekly injections, and one monthly injection. In their series of 40 patients and 61 eyes, clinical remission was achieved after a median of six injections, and recurrent ocular disease was successfully controlled with an additional treatment course. Unlike the protocol described by Zhou et al. [7], treatment in our cohort did not necessarily end after the initial seven injections. Continuation, temporary interruption, or resumption was individualized according to clinical findings, OCT evidence of active disease, relapse, and treatment tolerability. The median total number of injections was therefore 16, with a range of 12–25. Our regimen is consequently more accurately described as reduced-intensity induction followed by response-guided continuation rather than as a uniformly shortened treatment course. The median of 16 injections is also comparable to the reduced treatment burden suggested by Habot-Wilner et al. [14]. In their 20-year experience, patients received a mean of 19 injections, although substantially fewer injections were generally required to achieve remission. The authors suggested that approximately 15 injections might provide similar ocular control with fewer adverse effects.

Nevertheless, the present study did not include a conventional-treatment control group. It therefore cannot establish that response-guided treatment is equivalent or superior to the conventional 25-injection regimen. Furthermore, the total number of injections was determined by the individual disease course. Patients with persistent or recurrent disease were more likely to receive additional injections, resulting in treatment-by-indication bias. This may explain the absence of an association between injection number and complete remission.

Three patients experienced ocular relapse at 8, 10, and 12 months after treatment initiation. All three regained ocular disease control after intravitreal MTX was resumed. The successful response to retreatment is consistent with the findings of Zhou et al., who reported a second remission after an additional course of intravitreal MTX in patients with recurrent ocular disease [7]. Although the observed relapse rate was relatively low, the 12-month follow-up period limits the evaluation of long-term disease control. In the study by Klimova et al. [15], relapse was frequent during extended follow-up, with a median time to first relapse of 31 months. Similarly, long-term studies [16,17,18] have demonstrated that ocular or CNS relapse may occur several years after the initial response. Thus, the present relapse rate should be interpreted as a 12-month estimate rather than evidence of sustained long-term remission.

Better baseline VA was associated with complete ocular remission in the exploratory univariate analysis. This finding may indicate that patients with better preserved visual function had less extensive retinal infiltration or structural damage and were consequently more likely to achieve complete clinical and imaging resolution. The baseline OCT score also showed a possible association with remission, although statistical significance was not reached. Kim et al. [10] similarly identified baseline VA and retinal or subretinal infiltration as predictors of subsequent visual outcome. Nevertheless, our analysis included only three patients who did not achieve complete remission. The resulting group imbalance and limited statistical power increase the uncertainty of the estimates. These findings should therefore be regarded as hypothesis-generating and require validation in larger cohorts.

Documented CNS lymphoma was present in 10 patients, all of whom received systemic therapy or radiotherapy. Conversely, all seven patients without documented CNS involvement were treated with intravitreal MTX alone. Central nervous system status and additional treatment therefore overlapped completely and could not be analyzed as independent factors. No statistically significant differences in ocular remission, relapse, VA, injection number, or time to remission were observed between patients with and without documented CNS involvement. However, these comparisons were substantially underpowered and should not be interpreted as demonstrating equivalence. Moreover, the ocular outcomes of patients with CNS lymphoma cannot be attributed exclusively to intravitreal MTX because systemic therapy or radiotherapy may also have contributed to ocular disease control.

The role of systemic therapy in patients with isolated ocular disease remains controversial [15,19,20,21]. Klimova et al. [15] reported that combined local and systemic therapy prolonged the time to first relapse, whereas other retrospective studies and pooled analyses have not consistently demonstrated that systemic treatment prevents CNS progression or improves overall survival [19,20]. Given the close biological relationship between VRL and PCNSL, local ocular control should not be considered equivalent to systemic disease control. Continued multidisciplinary surveillance, including neurological assessment and appropriate neuroimaging, remains necessary even after complete ocular remission [21].

The ocular safety profile was manageable, although 70.6% of patients experienced at least one adverse event. Keratopathy was the most frequent complication, occurring in 41.2%, followed by transient elevation of intraocular pressure and conjunctival hyperemia. All adverse events were managed conservatively or by extending the interval between injections, and no patient permanently discontinued treatment. No cases of endophthalmitis, retinal detachment, or intraocular hemorrhage were observed. Keratopathy is the most consistently reported adverse effect of intravitreal MTX and appears to be related to injection frequency and cumulative exposure. Zhou et al. [8] reported keratopathy in 66% of treated eyes and observed a reduction from 86.4% with the conventional regimen to 25.0% after injection frequency was reduced. In the modified seven-injection protocol described by Zhou et al., corneal epitheliopathy occurred in only 3 of 61 treated eyes [7]. The keratopathy rate in our cohort was therefore lower than that reported with intensive conventional treatment but higher than that observed with the seven-injection regimen. This intermediate rate is consistent with the median exposure of 16 injections and suggests that reducing induction intensity may limit, but not eliminate, ocular surface toxicity. Regular examination of the corneal epithelium and adjustment of injection frequency remain important components of intravitreal MTX management [18,22].

Intravitreal MTX remains one of the most established local treatments for VRL, providing high intraocular drug concentrations while avoiding the systemic toxicity associated with high-dose intravenous chemotherapy. Early experience with intravitreal MTX demonstrated that ocular disease could be effectively controlled with a conventional intensive induction-consolidation-maintenance regimen, with remission generally achieved after several injections. Fishburne et al. [23] reported that clinical remission was achieved after a mean of 6.4 injections, while 95% of eyes were cleared of malignant cells after 13 injections or fewer. Subsequent studies have confirmed the efficacy of intravitreal MTX, although reported treatment schedules and response criteria have varied considerably between institutions [6,24,25,26]. In a 20-year retrospective series of 129 patients, intravitreal MTX was associated with effective control of vitreoretinal disease, further supporting its role as a cornerstone of local therapy [14]. However, the absence of a standardized dosing schedule remains an important limitation in the current literature. Most published protocols were developed empirically and involve a relatively high number of injections, potentially resulting in unnecessary cumulative exposure once complete ocular remission has been achieved. In this context, a response-adapted approach, in which the frequency of injections is modified according to objective clinical response and treatment-related toxicity, may represent a more individualized strategy [27,28]. Our findings therefore add to the growing body of evidence suggesting that treatment intensity can potentially be tailored to disease activity rather than following a fixed injection schedule.

The efficacy of intravitreal MTX should also be interpreted in the context of alternative intravitreal therapies, particularly rituximab. Intravitreal rituximab provides a biologically distinct approach through targeting of the CD20 antigen expressed by most VRL cells [29]. In a multicenter retrospective study, intravitreal rituximab with or without MTX achieved complete remission in 64.6% of treated eyes after a median of three injections, with partial remission in a further 22.9% [30]. Rituximab has also been investigated specifically in patients who developed severe MTX-associated corneal epitheliopathy, suggesting a potential role as an alternative or sequential treatment when MTX toxicity becomes clinically significant [31]. Nevertheless, the available evidence does not establish superiority of rituximab over MTX, and treatment approaches remain highly heterogeneous. Rather than viewing the two agents as mutually exclusive, they may be considered complementary components of local therapy, particularly in patients with recurrent disease or significant MTX-related toxicity. Importantly, neither intravitreal MTX nor rituximab addresses the underlying risk of CNS dissemination. Vitreoretinal lymphoma has a strong biological and clinical association with primary CNS lymphoma, and ocular clearance should therefore not be interpreted as systemic disease control [13,32].

Local therapy should be considered within the broader management of VRL. Ocular radiotherapy provides effective local control but is limited by radiation-related complications, while systemic high-dose MTX can treat CNS disease at the cost of greater systemic toxicity [13,19,33]. A recent meta-analysis found that ocular or combined treatment was associated with a lower risk of ocular relapse than systemic therapy alone, without demonstrating a clear overall survival benefit from adding systemic therapy [20]. Thus, treatment should be individualized according to ocular disease, CNS involvement, patient characteristics, and treatment-related toxicity. A response-adapted intravitreal MTX strategy may help maintain the efficacy of intravitreal chemotherapy while reducing cumulative treatment burden and toxicity. Unlike conventional fixed regimens, treatment intensity can be adjusted according to clinical response, with multimodal imaging and potentially intraocular IL-10 levels providing additional markers of disease activity [21,34,35]. Given the predominantly retrospective and heterogeneous nature of the existing literature, prospective studies are needed to establish whether response-adapted therapy can achieve sustained disease control with fewer injections and reduced toxicity. Until then, this approach represents a rational strategy for balancing efficacy, safety, and treatment burden.

Future research should prioritize prospective, multicenter studies comparing response-guided intravitreal MTX protocols with conventional fixed regimens to determine whether reduced induction intensity can maintain ocular disease control while decreasing treatment burden and cumulative corneal toxicity [7,8,14,18,22]. Standardized response criteria combining clinical examination with quantitative OCT parameters are also needed to distinguish active lymphoma from persistent post-treatment structural abnormalities [11,12,13,34,36]. In addition, minimally invasive molecular monitoring using aqueous humor biomarkers, including IL-10, MYD88 L265P, and cell-free tumor DNA, may facilitate earlier detection of treatment response and ocular relapse [35,37,38,39]. Finally, studies with longer follow-up should evaluate ocular and CNS outcomes separately and determine whether integration of local therapy with systemic treatment can reduce CNS progression without exposing patients with isolated ocular disease to unnecessary systemic toxicity [19,20,21]. These approaches may support the development of individualized treatment algorithms based on disease activity, molecular response, imaging findings, and the risk of CNS involvement.

The strengths of this study include the enrollment of consecutive patients treated at two ophthalmology centers, cytological confirmation supported by flow cytometry, use of a standardized initial treatment protocol, and complete 12-month follow-up. Functional, clinical, and OCT outcomes were evaluated at predefined time points. In patients with bilateral disease, selecting one study eye per patient avoided the statistical dependence associated with including both eyes in the analysis. Several limitations should be acknowledged. The retrospective design and small sample size increased the potential for selection bias, incomplete documentation, and imprecise effect estimates. Requiring a minimum follow-up of 12 months may also have excluded patients who were lost to follow-up early or experienced severe systemic deterioration. Furthermore, the absence of a comparator group precluded direct evaluation of the efficacy and safety of the response-guided protocol relative to the conventional 25-injection regimen. Treatment was individualized after the initial phase, resulting in variable treatment exposure and potential confounding by disease severity. CNS information was collected retrospectively, and the temporal relationship between ocular presentation and CNS diagnosis could not be reliably reconstructed. Consequently, CNS progression could not be evaluated. In addition, CNS involvement overlapped completely with the administration of systemic therapy or radiotherapy, preventing assessment of their independent effects on ocular outcomes. Optical coherence tomography findings were assessed qualitatively, without standardized quantitative measurements or masked image grading. Persistent abnormalities could therefore not be definitively classified as residual disease or inactive structural sequelae. Finally, the exploratory analysis of factors associated with complete remission was limited by the small number of nonresponders. These findings should therefore be regarded as hypothesis-generating rather than confirmatory.

## 5. Conclusions

A reduced-intensity induction regimen followed by response-guided continuation of intravitreal MTX was associated with a high rate of complete ocular remission, progressive improvement in VA, and a manageable safety profile over 12 months. Vitritis and retinal infiltrates resolved more consistently than structural OCT abnormalities, suggesting that persistent outer retinal changes after clinical remission may represent residual anatomical damage rather than active disease. However, because most patients with documented CNS involvement also received systemic therapy or radiotherapy, the observed outcomes cannot be attributed exclusively to intravitreal MTX. Larger prospective studies with standardized response criteria, appropriate comparison groups, and longer follow-up are required to confirm these findings and establish the optimal treatment schedule.

## Figures and Tables

**Table 1:** Baseline Clinical and Treatment Characteristics of the Study Cohort.

Parameter	Value
Patients, *n*	17
Enrollment period	2018–2023
Follow-up duration	12 months (all patients)
Intravitreal MTX (400 μg/0.1 mL)	17 (100%)
Total injections, median (range)	16 (12–25)
Additional concomitant therapy	10 (58.8%)
Intravitreal therapy alone	7 (41.2%)
Complete ocular remission within 12 months	14 (82.4%)
Did not achieve complete ocular remission	3 (17.6%)
Ocular relapse within 12 months	3 (17.6%)

Abbreviation: MTX, methotrexate. Nine patients received concomitant systemic therapy and one received radiotherapy. Remission status at 12 months and relapse during follow-up are distinct outcome measures.

**Table 2:** Evolution of clinical and OCT findings from baseline to 12 months.

Finding	Baseline, *n* (%)	12 Months, *n* (%)	*p* (McNemar)
Vitritis	16 (94.1%)	0 (0%)	<0.001
Retinal infiltrates	12 (70.6%)	0 (0%)	<0.001
Sub-RPE lesions	13 (76.5%)	7 (41.2%)	0.031
Outer retinal hyperreflectivity	12 (70.6%)	6 (35.3%)	0.031
Outer retinal disorganization	10 (58.8%)	7 (41.2%)	0.250
Foveal involvement	8 (47.1%)	7 (41.2%)	1.000
Subretinal fluid	1 (5.9%)	1 (5.9%)	1.000

*p*-values are unadjusted values from seven paired McNemar tests comparing baseline with the scheduled 12-month assessment and are presented for exploratory purposes. The Bonferroni-adjusted significance threshold is *p* < 0.0071; only the reductions in vitritis and retinal infiltrates met this threshold. Abbreviations: OCT, optical coherence tomography; RPE, retinal pigment epithelium.

**Table 3:** Exploratory univariate analysis of baseline and treatment-related factors associated with complete ocular remission.

Factor	Statistical Test	*p*-Value
Baseline VA (logMAR)	Mann–Whitney U	0.047
Baseline OCT score	Mann–Whitney U	0.067
Total MTX injections	Mann–Whitney U	0.945
Additional concomitant therapy	Fisher’s exact	0.228

*p*-values are univariate and exploratory. Because only three patients did not achieve complete remission, these analyses were underpowered and should not be interpreted as predictive. Abbreviations: VA, visual acuity; logMAR, logarithm of the minimum angle of resolution; OCT, optical coherence tomography; MTX, methotrexate.

**Table 4:** Ocular safety outcomes during intravitreal MTX therapy.

Adverse Event	*n*	%
Any ocular adverse event	12	70.6
Keratopathy	7	41.2
Transient intraocular pressure elevation	3	17.6
Conjunctival hyperemia	2	11.8
No ocular adverse event	5	29.4

Percentages were calculated using *n* = 17 as the denominator. Patient-level overlap among individual adverse-event categories was unavailable in the aggregated dataset. Abbreviation: MTX, methotrexate.

## Data Availability

The data presented in this study are available on request from the corresponding author, subject to institutional data-protection restrictions.
